# Effects of transcranial direct current stimulation combined with motor relearning program on strength and balance in stroke patients

**DOI:** 10.7717/peerj.18925

**Published:** 2025-02-19

**Authors:** Muhammad Hamad Haleem, Mirza Obaid Baig, Turki Abualait, Woo-Kyoung Yoo, Sumaiyah Obaid, Shahid Bashir

**Affiliations:** 1 Faculty of Rehabilitation & Allied Health Sciences, Riphah International University, Islamabad, Islamabad, Pakistan; 2 National Excellence Institute, Islamabad, Pakistan; 3 College of Applied Medical Sciences, Imam Abdulrahman Bin Faisal University, Dammam, Saudi Arabia; 4 Department of Physical Medicine & Rehabilitation, Sacred Heart Hospital, Hallym University, Anyang, Republic of South Korea; 5 Neuroscience Center, King Fahad Specialist Hospital, Dammam, Saudi Arabia; 6 King Salman Center for Disability Research, Riyadh, Saudi Arabia

**Keywords:** Balance, Stroke, Muscle strength, Transcranial direct current stimulation

## Abstract

**Background:**

A stroke is characterized by neurological deficits that result in compromised muscle strength and balance, impacting the overall wellbeing of the patient, including decreased quality of life, socialization and participation in daily activities. The aim of the study is to determine the effects of transcranial direct current stimulation combined with a motor relearning program on strength and balance in sub-acute stroke patients.

**Methods:**

The randomized controlled trial involved 44 subacute stroke patients, randomly assigned to either the experimental group (*n* = 22) or control group (*n* = 22). The intervention included anodal transcranial direct current stimulation (tDCS) for the experimental group and sham stimulation with a motor relearning program for the control groups. Assessments were conducted using manual muscle testing for muscle strength and the Berg Balance Scale for balance at baseline, the fourth week, and the eighth week.

**Results:**

There were no statistically significant effects in the experimental group for either strength or balance (*p*-value > 0.05) but there were time effects for both variables especially during the intervention period in both the experimental and control groups.

**Conclusion:**

There does not appear to be any short term or long-term additional effects of anodal transcranial direct current stimulation on strength and balance in subacute stroke patients.

## Introduction

A stroke can lead to multiple complications depending on the location and severity of the lesion, including altered consciousness, motor deficits, visual field deficits, aphasia, dysarthria, dysphagia, urinary incontinence (Saini, Guada & Yavagal, 2021), musculoskeletal difficulties, psychosocial complications, and late medical complications (Chohan, Venkatesh & How, 2019) with a noticeable symptoms of muscle weakness, which is also a key factor in the recovery of normal physical abilities (Bohannon, 2007; Cramp et al., 2006; Cauraugh & Kim, 2003), affecting about 65% of patients after a stroke (Wist, Clivaz & Sattelmayer, 2016). This can result in abnormal posture, weakening or loss of stretch reflexes and an inability to perform voluntary movements (Veerbeek et al., 2014).

Progressive resistance training and task specific training have the best evidence for improving muscle strength in stroke patients, although other interventions can also be beneficial (Wist, Clivaz & Sattelmayer, 2016). Similarly, exercise therapy, repetitive task training, physical fitness training, virtual reality (VR) and the use of unstable surfaces can be utilized to improve balance post stroke (Arienti et al., 2019).

It is common knowledge that neurons are excitable cells, and the production of action potentials is essential to their functionality. When the depolarization of the resting membrane surpasses a specific threshold, action potentials are produced. Direct current stimulation is used to modify neuronal resting potentials, which changes the excitability of the cell and increases the likelihood that an action potential with the appropriate magnitude will be generated. A direct current can depolarize a neuronal membrane, resulting in less afferent activity needed to trigger an action potential. Conversely, a hyperpolarized neuronal membrane will have less excitability and spontaneity. Anodal transcranial direct current stimulation (tDCS) is excitatory because it makes neuronal membranes more excitable, but cathodal tDCS is inhibitory because it deepens the resting membrane potential, making it more difficult to stimulate (Stagg, Antal & Nitsche, 2018). tDCS induces effects by improving regional cerebral blood flow, facilitating synaptic efficacy, and promoting the expression of neurotrophic factors like brain derived neurotrophic factor (BDNF) and tropomyosin receptor kinase B (TrkB) activation (Schjetnan et al., 2013). tDCS has been shown to have beneficial effects on mobility, muscle strength, motor learning, lower limb function, balance, gait, functionality, and walking ability in post stroke patients (Comino-Suárez et al., 2021; Saeys et al., 2015; Sohn, Jee & Kim, 2013; Elsner et al., 2020; Navarro-Lopez et al., 2021) and appears to be a promising intervention for stroke patients. However, it is still in the experimental phase and further research is required to confidently use tDCS in everyday practice.

Salazar et al. (2020) found mild improvements in handgrip strength using bicephalic tDCS and functional electrical stimulation (FES) in patients with moderate to severe chronic stroke. They also found significant improvement in grip strength when combined with occupational therapy (Mortensen, Figlewski & Andersen, 2016) and modified constrained induced movement therapy (mCIMT) (Rocha et al., 2016). Another study similarly found increased grip strength in chronic patients when using tDCS and long term effects of tDCS on handgrip in patients with subacute stroke (Cha et al., 2014). A pilot study by Geroin et al. (2011) used tDCS combined with robot assisted gait training and found improvements in muscle strength of the lower leg in chronic stroke patients. Khedr et al. (2013) found improvements in handgrip strength, shoulder abduction, dorsiflexion, hip flexion and quadriceps when using anodal tDCS in patients with subacute stroke (Sohn, Jee & Kim, 2013). Chang, Kim & Park (2015) have shown that anodal tDCS supplements the effects of conservative physical therapy for lower extremity strength in patients with subacute stroke. However, some authors did not find any significant improvements in upper limb and lower limb muscle strength when using tDCS in combination with conventional therapy or active tDCS compared to sham stimulation (Andrade et al., 2017; Hesse et al., 2011; Fusco et al., 2014).

Tanaka et al. (2011) concluded that anodal tDCS has modest transient effects on knee extensor strength and does not affect the maximal handgrip force. Similarly, Palimeris et al. (2022) found no significant improvements in strength, Geiger et al. (2019) found no improvements in the strength of knee extensors in chronic stroke. A meta-analysis by Sun et al. (2021) on the effects of electrical stimulation on strength in stroke patients included only one study that had a population of subacute stroke. The mixed literature on the evidence of tDCS influence on strength in subacute stroke highlights the dearth of literature on subacute stroke. Additionally, there has been little literature available on the subacute stage of stroke and the available literature focused on immediate and short-term goals. We only incorporated the concept of a newly published protocol of repetitive transmagnetic stimulation, the Stanford Accelerated Intelligent Neuromodulator Therapy (SAINT) among depression (Cole et al., 2020), rather than implementing all of its parameters. The stimulation session were adjusted to occur twice as frequently as usual in order to observe any effect, as the wide variety of protocols used may be contributing to the inconsistent results obtained with tDCS. Therefore, this study aimed to investigate the long-term effects of tDCS in the subacute stage of stroke.

## Methods and Materials

### Participants

The study protocol was approved by the Research Ethical Committee of Riphah International University (RIPHAH/RCRS/REC/Letter-01355), registered with ClinicalTrials.gov (ID: NCT05878626), conformed to the CONSORT checklist and was conducted between May 20th and November 30th, 2023, at Rafsan Rehabilitation Center in Peshawar, Pakistan. The experiment was conducted in accordance with the Helsinki Declaration. After obtaining the written consent from the participants, data on demographics and stroke diagnosis, type, site and onset were collected using a demographic questionnaire and medical charts. The inclusion criteria were (a) individuals aged 45–70 years of both genders (no females were included due to the rehabilitation center being located in a conservative area where the majority of patients are males from tribal areas of Khyber Pakhtunkhwa), (b) ischemic stroke with either cortical or subcortical involvement, (c) subacute stroke (one week to six months post stroke), and first MCA stroke, (d) medium to high fall risk on the Berg Balance Scale (BBS). The exclusion criteria were (a) hearing and visual deficits, (b) patients with other neurological conditions such as multiple sclerosis or Parkinson disease, (c) skull wounds, (d) presence of shunts or metallic implants in the skull, (e) brain tumors, (f) musculoskeletal conditions affecting the lower limbs, (g) cognitive impairment.

### Experimental design

**Figure 1:**
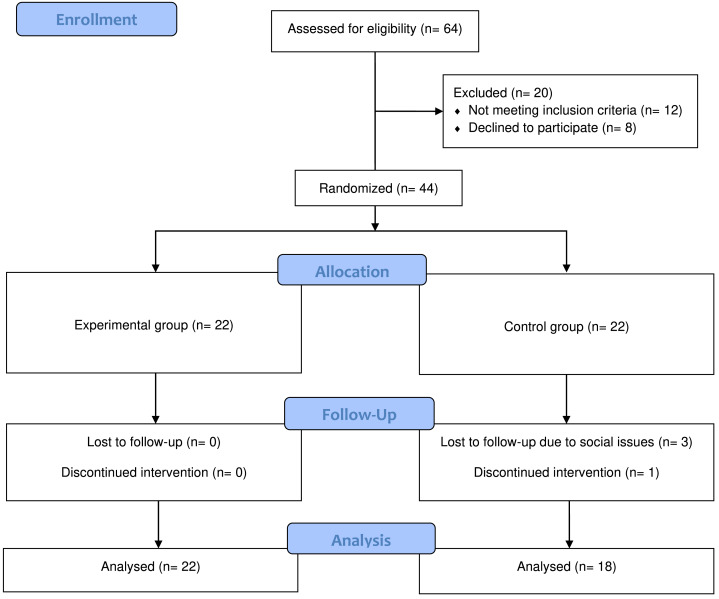
CONSORT diagram.

This study was a double blinded (patient and assessor blind) randomized controlled trial with pre-, post- and one month after intervention assessments. A third person from the center, not directly involved with patients and not a physical therapist randomly generated allocations within opaque sealed envelopes. The envelopes were signed and sequentially numbered. The envelope remained sealed until the patient gave their consent to participate in the study. The envelope was then opened, and the patient was allocated accordingly to either the experimental or control group. The experimental group received active tDCS with conventional therapy and the control group received sham tDCS with conventional therapy twice before and after the conventional therapy five days a week for four weeks. Refer to Fig. 1 for the experimental flow.

### Intervention

Active anodal transcranial direct current stimulation (a-tDCS) was applied using 1 ×1 low intensity transcranial electrical stimulator manufactured by Soterix Medical. This stimulation was administered twice, with a 30-minute interval to the M1 area of participants for 20 min at an intensity of 2 mA, using a 35 cm^2^ (5×7 cm) electrode. Due to the large size of tDCS electrodes (5 ×7 cm), the anodal electrode covered the multiple areas of M1 including hand, arm, trunk and the lower extremity (Cuthbert & Goodheart Jr, 2007). The design of this study was set up with the assumption that primary motor cortex (M1) has reciprocal dense connections with cerebellum and other cortical regions (Cuthbert & Goodheart Jr, 2007; Nitsche & Paulus, 2000). Convergence evidence has shown that such connections are critical in performing activities of daily living and refining motor learning (Pascual-Leone et al., 1995; Siebner & Rothwell, 2003; Ugawa et al., 1995; Pinto & Chen, 2001). A systematic review and meta-analysis study revealed that tDCS might be an effective option for restoring gait and recovering motor functional mobility for patients with stroke (Yu et al., 2007). Therefore, stimulating M1 might probe this pathway and enhance motor functional recovery in patients with stroke.

The M1 stimulation point was identified by measuring the distance from the glabella to the inion, and then the distance between the supra-auricular areas of both ears to the superior point of the skull. The intersection of these lines is the vertex of the skull. The M1 point, corresponding to C3 and C4 on the EEG 10/20 system, was located by traveling down 20% of the length of the line from supra-auricular area (Spampinato, Celnik & Rothwell, 2020). Sham stimulation was administered by selecting the sham option on the unit. Conventional treatment consisted of a motor relearning program where patients were instructed to repeatedly perform activities listed in Table 1. This treatment was provided by two physiotherapists with master’s level formal training in physical therapy and two years of experience working with stroke patients. The physiotherapeutic intervention lasted for 30 min between the two stimulations, as outlined in Table 1.

**Table 1:** Motor relearning program (MRP).

Week 1 & 2	Reach forwards in sitting
	Reach sideways in sitting
	Reach backwards in sitting
	Step forwards with unaffected limb
	Step backwards with unaffected limb
	Sitting resisted knee extension
	Repeat same activities with unaffected limb in sitting
	Supported standing
	Unsupported standing
	Standing, look up and return to mid position
	Standing, turn head and body behind and return to mid position
	Heel raise and lower
Week 3 & 4	Standing reach forwards, sideways and backwards
	Step forwards with unaffected limb
	Step backwards with unaffected limb
	Step up with affected limb
	Step down with unaffected limb
	Forward step downs with unaffected limb
	Lateral step ups with unaffected limb
	Walking up a ramp
	Sitting resisted knee extension

### Outcome measures

Manual muscle testing (MMT) was used as an outcome tool to assess the strength of lower limb muscles while the BBS was used to assess balance at weeks zero, four and eight.

#### MMT

MMT is utilized to determine the extent and degree of muscular weakness resulting from disease, injury, or disuse. It is a crucial part of assessment in various patient groups including those with stroke, spinal cord injury, neuropathy, and other neurological and musculoskeletal conditions. Patients can be scored on a scale of 0–5, with grade 5 indicating completion of the full range of motion (ROM) against maximum resistance from the therapist, grade 4 indicating completion of full ROM against moderate resistance, grade 3 indicating completion of full ROM against gravity, grade 2 indicating completion of ROM with gravity eliminated, grade 1 indicating flickering of muscles when movement is attempted and grade 0 indicating no palpable contraction or flickering. A review on the validity and reliability of MMT reported ICC values of up to 0.96 suggesting it is a reasonably valid tool to assess muscle strength (Dong et al., 2021). The muscles tested using MMT included hip extensors, knee extensors, ankle plantar flexors and ankle dorsi flexors.

#### BBS

The BBS is an objective assessment of a patient’s ability to safely balance while performing a series of specified exercises. A five-point scale from 0 to 4 is used for each of the 14 items on the list, with 0 denoting the lowest level of function and four denoting the highest level of function. The maximum score is 56, indicating normal function. Scores of 41 to 56 indicate a mild risk of falling, 21 to 40 indicate a medium risk of falling, and 0 to 20 indicate a high risk of falling. A study on various scales used for assessing balance and function reported ICC values of 0.99 (DaSilva et al., 2011).

The BBS was administered by asking the patient to perform the 14 activities mentioned in the questionnaire, with each activity scored on a scale of 0–4, where 0 indicated the lowest level of that activity and four indicated the highest level.

### Sample size

For sample size calculation, G*power version 3.1.9.4 (https://www.psychologie.hhu.de/arbeitsgruppen/allgemeine-psychologie-und-arbeitspsychologie/gpower) was used. F tests ANOVA repeated measures were selected as this study included repeated measurements. The effect size was set to 0.25 resulting in a sample size of 44 patients divided into two groups of 22 participants each.

### Statistical analysis

Data analysis was conducted using SPSS version 27 employing descriptive and inferential statistics. The normality of variables was assessed using the Shapiro–Wilk test for participants who completed the study protocols. All demographic variables were found to be not-normally distributed, leading to the application of the Chi-square test to identify differences between the two groups at baseline. The variables MMT (hip extensors, knee extensors, ankle plantar flexors, and ankle dorsiflexors) were also non-normally distributed, prompting the use of the Mann–Whitney U Test, Friedman test and Wilcoxon signed-rank test for between-group, within group and post hoc analyses respectively. On the other hand, The BBS scores were normally distributed, allowing for the utilization of independent *t*-test, ANOVA and Bonferroni tests for between-group, within group and post hoc analyses respectively. A *p*-value of <0.05 was deemed significant.

## Results

The mean age of participants in both groups was 56.80 ± 4.32 years, with the experimental group being 57.18 ± 4.65 and the control group being 56.41 ± 4.04 years. The average duration of stroke onset was 3.61 ± 1.41 months with 63.6% being right hemiplegic. Detailed characteristics of participants are available in Table 2.

**Table 2:** Characteristics of participants.

Variable		Overall	EG (*n* = 22)	CG (*n* = 22)	*P* value
Age (years)		56.80 ± 4.327	57.18 ± 4.656	56.41 ± 4.043	.523
Gender	Males (n)	44	22	22	
	Females (n)	0	0	0	
Site of involvement	Right hemiplegia (n) (%)	28 (63.6%)	9 (40.91%)	19 (86.36%)	.002
	Left hemiplegia (n) (%)	16 (36.4%)	13 (59.09%)	3 (13.64%)	
Comorbid	HTN (n) (%)	31 (70.5%)	19 (86.36%)	12 (54.55%)	.103
	DM (n) (%)	1 (2.3%)	0	1 (4.55%)	
	HTN and DM (n) (%)	10 (22.7%)	3 (13.64%)	7 (31.82%)	
	Nil (n) (%)	2 (4.5%)	0	2 (9.09%)	
Nature of work	Driver (n) (%)	8 (18.2%)	3 (13.64%)	5 (22.73%)	.804
	Shopkeeper (n) (%)	5 (11.4%)	3 (13.64%)	2 (9.09%)	
	Labor (n) (%)	13 (29.5%)	8 (36.36%)	5 (22.73%)	
	Retired (n) (%)	7 (15.9%)	3 (13.640	4 (18.18%)	
	Others (n) (%)	11 (25.0%)	5 (22.73%)	6 (27.27%)	
Duration of stroke (months)		3.61 ± 1.418	4.00 ± 1.195	3.23 ± 1.543	.366

### Manual muscle testing

Since all components of MMT were not-normally distributed, the Mann–Whitney U test was used to compare means between groups. The mean rank value for hip extensors (HE) after four weeks of intervention was 21.77 in the experimental group and 22.24 in the control group with a *p*-value of 0.892. The mean ranks in knee extensors (KE) were 20.07 and 24.02, *p*-value = 0.274. The mean ranks for ankle plantar flexors (PF) were 19.74 and 24.38, *p*-value = 0.208 for the experimental and control group, respectively. The mean rank values of ankle dorsiflexors (DF) were 18.95 for the experimental group and 25.19 for the control group, *p*-value = 0.089. The analysis indicates that there were no statistically significant effects of a-tDCS in the experimental group. There were no significant effects after one month of follow-up between all the components of MMT (Table 3).

**Table 3:** Between group analysis—Mann–Whitney U test for MMT.

**Variable**	**Group**	**Mean ± S.D**	**Mean Rank**	***P*-value**
Hip Extensors at 0 weeks	Experimental	2.98 ± .762	18.68	0.32
	Control		26.32	
Hip Extensors at 4th week	Experimental	3.44 ± .666	21.77	0.892
	Control		22.24	
Hip Extensors at 8th week	Experimental	3.28 ± .716	20.32	0.905
	Control		20.72	
Knee Extensors at 0 weeks	Experimental	2.77 ± .859	18.91	0.049
	Control		26.09	
Knee Extensors at 4 weeks	Experimental	3.05 ± .844	20.07	0.274
	Control		24.02	
Knee Extensors at 8 weeks	Experimental	2.83 ± .781	21.50	0.497
	Control		19.28	
Ankle Plantar Flexors 0 weeks	Experimental	1.84 ± 1.098	19.45	0.106
	Control		25.55	
Ankle Plantar Flexors at 4 weeks	Experimental	2.56 ± 1.161	19.73	0.208
	Control		24.38	
Ankle Plantar Flexors at 8 weeks	Experimental	2.17 ± 1.035	19.82	0.666
	Control		21.33	
Ankle Dorsi Flexors at 0 week	Experimental	1.98 ± 1.023	17.98	0.105
	Control		27.02	
Ankle Dorsi Flexors at 4th week	Experimental	2.35 ± .973	18.95	0.089
	Control		25.19	
Ankle Dorsi Flexors at 8th week	Experimental	1.95 ± 1.011	19.36	0.471
	Control		21.89	

For within group analysis of all components of MMT, the Friedman test was used. The results of the Friedman test showed an increase in the grade of MMT within the same group for both groups for all components of MMT (*p* ≤ 0.05) (Table 4). The table suggests that similar strength in both ankle dorsiflexors and plantarflexors might be due to dorsiflexors being tested in the short sitting position which may have allowed a mechanical advantage to dorsiflexors resulting in similar strength values to plantarflexors. For post hoc analysis, the Wilcoxon signed-rank test was used (Table 5). The Wilcoxon signed-rank test shows a positive effect on MMT from zero weeks to the 4th week for all components of MMT in the experimental group (*p* ≤ 0.05).

**Table 4:** Within group analysis—Friedman test for MMT.

Stage	Mean Rank	Mean ± SD	*p* value	Mean Rank	Mean ± SD	*p* value
	**Experimental**			**Control**		
Hip Extensors						
0 week	1.52	2.73 ± .767	0.001	1.81	3.17 ± 0.707	.022
4 weeks	2.36	3.41 ± .734		2.22	3.44 ± .616	
8 weeks	2.11	3.27 ± .767		1.97	3.28 ± .669	
Knee Extensors						
0 week	1.64	2.50 ± .859	0.004	2.11	3.06 ± .802	0.006
4 weeks	2.20	2.91 ± .811		2.28	3.17 ± .857	
8 weeks	2.16	2.91 ± .684		1.61	2.72 ± .895	
Ankle Plantar Flexors						
0 week	2.18	2.14 ± 1.207	0.001	1.75	2.22 ± .808	0.001
4 weeks	1.41	1.50 ± 1.300		1.64	2.17 ± .707	
8 week	2.41	2.27 ± 1.420		2.61	2.83 ± .707	
Ankle Dorsi Flexors						
0 week	2.32	2.09 ± .868	0.004	2.39	2.56 ± 1.042	0.016
4 weeks	2.05	1.91 ± 1.151		1.64	2.00 ± .840	
8 weeks	1.64	1.59 ± .959		1.97	2.22 ± .943	

**Table 5:** *Post-hoc* analysis—Wilcoxon signed-rank for MMT.

Variable	Pairs		*p*-value	
Hip Extensors	0 week×8 weeks	EG	0.15
		CG	.157
	0 week×4 weeks	EG	<0.01
		CG	.014
	4 weeks×8 weeks	EG	.366
		CG	.083
Knee Extensors	0 week×8 weeks	EG	0.14
		CG	0.34
	0 week×4 weeks	EG	.003
		CG	.180
	4 weeks×8 weeks	EG	1.000
		CG	.005
Ankle Plantar Flexors	0 week×8 weeks	EG	.005
		CG	.705
	0 week×4 weeks	EG	<.001
		CG	<.001
	4 weeks×8 weeks	EG	.317
		CG	.002
Ankle Dorsiflexors	0 week×8 weeks	EG	0.52
		CG	.206
	0 week×4 weeks	EG	.002
		CG	.058
	4 weeks×8 weeks	EG	1.57
		CG	.012

### Berg balance scale

The values on BBS were normally distributed, so an independent *t*-test was applied to compare the means. No statistically significant effects existed between the two groups at zero weeks, four weeks and eight weeks (*p* ≥ 0.05) (Table 6).

**Table 6:** Between group analysis—Independent *t*-test for BBS.

Variable			Mean ± SD		*p*-value
0 week	Experimental	27.27 ± 5.496	.924
	Control	27.45 ± 6.913	
4 week	Experimental	30.91 ± 6.156	.723
	Control	31.67 ± 7.729	
8 week	Experimental	30.55 ± 6.759	.869
	Control	30.94 ± 8.412	

For within group analysis of BBS, repeated measures ANOVA was used (Table 7), showing a statistically significant difference between the three assessments in both groups (*p*-value ≤ 0.001) (Table 8). A statistically significant change was seen between pre and post intervention on post hoc analysis, but not between post intervention and one month of follow-up (Tables 9 and 10).

**Table 7:** Equality of error variances.

**Variable**	**Levene’s statistics**	***p*-value**
0 week	2.309	.136
4 week	2.142	.151
8 week	1.957	.170

**Table 8:** Within group analysis—repeated measures ANOVA for BBS.

**Variable**	**Group**	**Mean**	**Std. Error**	***p*-value**
0 week	EG	27.273	1.172	<0.01
	CG	28.056	1.731	
4 weeks	EG	30.909	1.312	
	CG	32.000	1.866	
8 weeks	EG	30.545	1.441	
	CG	30.944	1.983	
Grand Mean	EG	29.576	1.267	
	CG	30.333	1.834	

**Table 9:** *Post-hoc* analysis for BBS.

*Post hoc* analysis				
	**Experimental**		**Control**	
	**Mean difference**	***p*-value**	**Mean difference**	***p*-value**
0 week*8 week	−3.273	<0.001	−2.889	.003
0 week*4 week	−3.636	<0.001	−3.944	<.001
4 week*8 week	−.364	1.000	−1.056	.251

**Table 10:** Two-way ANOVA for BBS.

Variable	Mean ± SD		*p*-value	df	F-value	Mean square	Partial Eta Squared
0 week	Experimental	27.27 ± 5.496	.924	1	.009	.364	.000
	Control	27.45 ± 6.913					
4 week	Experimental	30.91 ± 6.156	.723	1	.127	6.166	0.003
	Control	31.67 ± 7.729					
8 week	Experimental	30.55 ± 6.759	.869	1	0.028	1.576	0.001
	Control	30.94 ± 8.412					

## Discussion

The objective of this study was to determine the effects of a-tDCS on strength and balance in subacute stroke patients. The results of the study showed that there were no statistically significant differences between the experimental group receiving a-tDCS and the control group receiving sham tDCS. However, within group analysis revealed statistically significant effects of a-tDCS on strength and balance in both the experimental and control group. *Post-hoc* analysis of MMT showed significant improvements between pre and post intervention periods, with inconsistent improvements between post intervention and follow up periods. Post hoc analysis of BBS also showed statistically significant improvements between pre and post intervention periods, but no improvements between post intervention and follow up periods.

It has been demonstrated that after a stroke there is a difference in cortical excitability between the two hemispheres of the brain (Alghadir et al., 2018). The hemisphere affected by the stroke is underactive, while the unaffected hemisphere becomes overactive. tDCS aims to restore the balance between the two hemispheres and improve overall brain function (Palimeris et al., 2022). Anodal tDCS improves cortical excitability in the affected hemisphere, while cathodal tDCS suppresses cortical excitability in the unaffected hemisphere (Stagg, Antal & Nitsche, 2018).

Klomjai et al. (2018) conducted a study on patients with subacute stroke, using a single session of dual tDCS with anodal stimulation on the ipsilesional side and cathodal stimulation on the contralesional side. They found that muscle strength did not change after the experimental treatment. Despite a significant difference in the number of sessions administered, the results of Klomjai et al. (2018) are consistent with our study. In another multicenter RCT on patients with subacute stroke, it was found that six weeks of intervention had no effect on upper limb strength, using an intensity of 2 mA for 20 min per session (Hesse et al., 2011). Sohn, Jee & Kim (2013) in their study on patients with subacute stroke administered one session each of active stimulation followed by sham stimulation after 48 h. They observed statistically significant improvements in knee extensor strength (Sohn, Jee & Kim, 2013), which differs from the findings of our study. Sohn, Jee & Kim (2013) included only 11 patients making it challenging to draw conclusions about the effectiveness of anodal tDCS in subacute stroke. However, their study provides immediate results of tDCS which are well documented in the literature. In a long-term study following patients with subacute stroke for 3 months, borderline improvements were reported in the group that received anodal stimulation (Khedr et al., 2013). These results contrast with our study possibly due to the fact that our study mainly included participants in the late subacute stage, where spontaneous recovery is slowing down or has stopped completely. Additionally, their study included only 14 patients who received anodal stimulation (Khedr et al., 2013).

Regarding the subacute stage of a stroke, anodal tDCS shows mixed results but the general trend is leaning towards no effect on strength. This aligns with the findings of our study (Tanaka et al., 2011). However, unlike Khedr et al. (2013) who observed superior results of tDCS stimulation (either anodal or cathodal) in enhancing the effects of rehabilitation training to improve motor recovery after a stroke, the current study did not find any improvement even after 2 months. This could be because Khedr et al. (2013) used TMS and/or faradic current stimulation in severe stroke cases. Chang, Kim & Park (2015) also noted improvements in lower limb muscle strength after administering anodal tDCS to subacute patients for 10 sessions, each lasting 10 min. In contrast, Rocha et al. (2016) found no significant results when using bicephalic tDCS to enhance grip strength over 12 sessions, applying anodal tDCS to the lesioned hemisphere and cathodal to the non-lesioned hemisphere. Similarly, Geroin et al. (2011) found no significant difference in lower leg strength between the anodal stimulation and control group in chronic stroke patients. Other studies have also reported no disparities in lower leg strength values. These findings align with our study results, despite the varying stages of stroke (Picelli et al., 2015; Seo et al., 2017).

Only Mortensen, Figlewski & Andersen (2016) demonstrated that five consecutive tDCS sessions, alongside conventional treatment improved grip strength in patients with hemorrhagic stroke. The difference in stroke types between Mortensen, Figlewski & Andersen (2016) and our study may have contributed to the difference.

There are very few studies that have studied strength as their primary variable and even fewer studies on the strength of lower limb muscles. However, the results of all these studies are mixed and no certain conclusion can be drawn. Variance in protocols, intensities, and montages used may contribute to these results. Accurate placement of electrodes is essential for the administration of tDCS as current distribution in the brain is highly dependent on electrode position.

The results of our study indicate that there were no statistically significant differences between the two groups after one month of intervention. Chang, Kim & Park (2015) reported similar results using 2 mA for 10 min once daily for 2 weeks on patients with subacute stroke, totaling 10 sessions. They found no significant difference in the tDCS group compared to sham stimulation (Chang, Kim & Park, 2015).

Another study on subacute stroke by Klomjai et al. (2018) also found no significant differences between the two groups. However, they only administered a single session of tDCS, and balance was measured using a different outcome tool than in our study. Sohn et al. reported improved balance with eyes open and eyes closed. The results contrast with ours, likely due to the low sample size of 11 and a very short intervention period (single session) (Sohn, Jee & Kim, 2013). Similarly, Danzl et al. (2013) found no differences between the groups post intervention and after one month of follow up. Their study included only eight patients with chronic stroke. Geroin et al. (2011) found no statistically significant differences between treatment and control groups on the Rivermead Mobility Index used for balance in patients with chronic stroke after 2 weeks of active tDCS stimulation. Madhavan et al. (2020) similarly found that there were no statistically significant differences for outcomes of balance between the groups that received active tDCS and conventional physical therapy with sham stimulation. The results of the above studies are consistent with the results of our study.

The apparent ineffectiveness of tDCS in improving balance after stroke can be misleading. Balance is controlled by several other factors such as cerebellar control, proprioception, and an intact somatosensory system. It seems that, similar to our study, the stimulation of the M1 motor area has no effect on the balance of stroke patients, regardless of the stage of stroke. This suggests that M1 stimulation might not be ideal for improving balance. Conversely, a wide variety of application techniques, protocols and montages may affect the effectiveness of tDCS resulting in no improvement in balance. This aspect of research on brain stimulation requires further exploration and understanding. The results of this study should be interpreted with certain limitations in mind. This was a single center trial involving only ischemic stroke patients, with no female participants. No objective measurements were taken at the brain level for neuroplasticity. No formal criteria were used for documenting the adverse effects of tDCS.

## Conclusions

Despite following a recently published protocol, there appear to be no short term or long-term effects of anodal tDCS on strength and balance in subacute stroke. Although there seems to be no difference between the two groups, within group analysis revealed that the two interventions were equally effective. Although not objectively measured, it can be concluded based on subjective observations that the patients tolerated the new protocol without any serious adverse effects that would necessitate stopping the intervention. As indicated by *post-hoc* analysis, improvements in strength were significant in the experimental group most of the time after four weeks of intervention.

##  Supplemental Information

10.7717/peerj.18925/supp-1 Supplemental Information 1 Protocol of the study

10.7717/peerj.18925/supp-2 Supplemental Information 2 CONSORT checklist

## Additional Information and Declarations

### Data Availability

The following information was supplied regarding data availability: The data is available at Figshare: Baig, Mirza Obaid (2024). Data.xlsx with Codes. figshare. Dataset. https://doi.org/10.6084/m9.figshare.27073171.v1.

### Clinical Trial Registration

The following information was supplied regarding Clinical Trial registration: NCT05878626
