# Supplementary material for: Effects of transcranial direct current stimulation combined with motor relearning program on strength and balance in stroke patients
Source: PeerJ. 2025 Feb 19;13:e18925. doi: 10.7717/peerj.18925 (PMC11846504; doi:10.7717/peerj.18925)
Supplement: Supplemental Information 1 [file peerj-13-18925-s001.docx]

EFFECTS OF TRANSCRANIAL DIRECT CURRENT STIMULATION COMBINED WITH MOTOR RELEARNING PROGRAM ON STRENGTH AND BALANCE IN STROKE PATIENTS

Introduction & Literature Review

According to WHO, stroke is defined as an accident to the brain with rapidly developing focal and global disturbances of cerebral functions with symptoms lasting 24 hours or longer or leading to death without any other apparent cause beside vascular origin including cerebral infarction(87%), cerebral hemorrhage(10%) and subarachnoid hemorrhage(3%)([1](#_ENREF_1)).

An estimate from WHO suggest 15 million people suffer stroke worldwide each year. 5 million die and 5 million are permanently disabled. In Pakistan, the incidence rate of stroke is 250 per 100,000([2](#_ENREF_2)). In the province of KP, the prevalence is 1.2% mostly affecting people younger than 50 years of age with comorbid obesity, hypertension, diabetes mellitus and smoking([3](#_ENREF_3)).

Stroke causes multiple complications including but not limited to loss of muscle strength, proprioception impairment, cognitive impairment, balance and gait impairments with increases risk of fall and decreases quality of life ([4-7](#_ENREF_4)). Balance is essential component of quality of walking and can be further broken down into 3 aspects: steadiness, symmetry and dynamic stability which are adversely affected in stroke([8](#_ENREF_8)). 80% of stroke survivors cannot maintain balance which affect walking ability of patients([9](#_ENREF_9)).

Loss of strength is a common complication post stroke which leads to loss of balance and walking ability. Variety of interventions are adopted to improve muscle strength after stroke, include progressive resistance training, specific task training or functional training, functional electrical stimulation and high intensity aerobic exercises ([10](#_ENREF_10)).

There are a variety of interventions to improve balance in post stroke patients. These include reaching exercises, functional task specific training, walking training like challenge to standing balance, providing visual and auditory feedback, progressive balance training, lower limb strengthening exercises and ankle foot orthoses([11-16](#_ENREF_11)). A meta-analysis has shown that improving the muscle strength of lower limb (hip extensors, knee extensors, ankle plantar flexors and dorsiflexors) has positive effects on balance in hemiparetic stroke patients ([10](#_ENREF_10)).

Transcranial direct current stimulation (tDCS) is a relatively new treatment approach for stroke recovery. tDCS is a neuromodulation method which involves application of weak direct current stimulations over the scalp via electrodes**.** It could induce plasticity via modulation of resting membrane potential and modification of spontaneous discharge rate([17](#_ENREF_17)). tDCS has been shown to have beneficial effects on mobility, muscle strength, motor learning, lower limb function, balance, gait, functionality and walking ability in post stroke patients([18-22](#_ENREF_18)). tDCS appears to be a promising intervention for stroke patients however its effects are not significant if used in isolation. Thus, it should be used as an adjunct to some other treatment.

Review of available literature indicates tDCS can be a good option in short and intermediate run but its effects in the long run have not been studied yet. Furthermore, to the best of my knowledge there is little literature available about the long-term effects of tDCS on muscle strength in subacute stages**.** Therefore, this study is designed to test the long-term effects of tDCS stimulation on subacute stroke patients.

**Objectives**

To determine the short and long term effects of anodal transcranial direct stimulation on lower limb muscle strength in subacute stroke.

To determine the short and long term effects of anodal transcranial direct stimulation on balance in subacute stroke.

**Material & Methods**

**Design**

The study is presented as a randomized controlled trial, an experimental scientific procedure characterized by the random distribution of the members of a suitable population group into an intervention group and a control group, comparing a specific impact between the groups after a period established in advance.

Its design is based on the measurement and comparison of the variables to be studied before and after the exposure of the participants to the experimental intervention and four weeks after the intervention.

**Participants**

Participants will be selected based on the following criteria:

**Inclusion criteria:**

- Subjects diagnosed with ischemic subacute MCA stroke of both genders between the ages of 45 and 60,
- Having medium to high risk fall as measured on BBS (BBS score ≤ 40)
- Consent signed by the patient (Annex I).

**Exclusion criteria:**

Subjects with:

- History of recurrent CVA
- Hearing and visual loss defects
- Wound at skull
- Presence of shunt or metallic implant at cranial region
- Brain tumors
- Musculoskeletal conditions affecting the lower limbs
- Cognitively compromised
- Suffering from other neurological conditions like MS, Parkinson’s etc

**Intervention:**

The intervention group will receive anodal tDCS with spongy electrodes applied to the M1 (supplementary motor area) of the skull which corresponds to C3 and C4 on the 10/20 EEG system. The intensity of TDCS will be 2.5 mA and the duration will be 20 mints. The intervention will be applied twice a day with a time difference of 30 minutes between the two sessions. Intervention will be withdrawn if the patient experience respiratory or motor paralysis with cramping of the hands accompanied by nausea, headache, blurred vision, pain or itching under the electrodes.

The conventional treatment in the form of a motor relearning program (MRP) (table 1).

**Table 1: Conventional Treatment (MRP) (**[**23**](#_ENREF_23)**,** [**24**](#_ENREF_24)**)**

| **Week 1 & 2** | 1. Reach forwards in sitting 2. Reach sideways in sitting 3. Reach backwards in sitting 4. Step forwards with unaffected limb 5. Step backwards with unaffected limb 6. Sitting resisted knee extension 7. Repeat same activities with unaffected limb in sitting 8. Supported standing 9. Unsupported standing 10. Standing, look up and return to mid position 11. Standing, turn head and body behind and return to mid position 12. Heel raise and lower |
| --- | --- |
| **Week 3 & 4** | 1. Standing reach forwards, sideways and backwards 2. Step forwards with unaffected limb 3. Step backwards with unaffected limb 4. Step up with affected limb 5. Step down with unaffected limb 6. Forward step downs with unaffected limb 7. Lateral step ups with unaffected limb 8. Walking up a ramp 9. Sitting resisted knee extension |

**Outcome Measures**

Sociodemographic variables: age, sex, educational level, type of work, time since onset, dominant side, affected side, past medical history, previous injury, chronic illness, pre stroke hypertension and diabetes, pre stroke adhesive capsulitis.

**Manual Muscle Testing (MMT)**

MMT is used to determine the extent and degree of muscular weakness resulting from disease, injury or disuse. It is an important part of assessment in many patient groups including patients with stroke, spinal cord injury, neuropathy and other neurological and musculoskeletal conditions. The patient can be scored by 5 grades where grade 5 is the patient completes full ROM against maximum resistance from therapist, grade 4 is patient completes full ROM against moderate resistance, grade 3 is patient completes full ROM against gravity, grade 2 is completion of ROM with gravity eliminated, grade 1 is flickering of muscles when movement is attempted and grade 0 is when there is no palpable contraction or flickering. A review on the validity and reliability of MMT reported ICC values of up to 0.96 suggesting it is a reasonably valid tool to assess muscle strength ([25](#_ENREF_25)).

**Berg Balance Scale (BBS)**

BBS is used to objectively determine a patient’s ability to safely balance during a series of predetermined tasks. It is a 14 item list with each item consisting of a five point scale ranging from 0 to 4, 0 indicating the lowest level of function and 4 indicating the highest level of function. The maximum score is 56 indicating normal function. A score of 41 to 56 indicates mild risk fall, 21 to 40 indicates medium risk fall and 0 to 20 indicates high risk fall. A study on various scales used for assessing balance and function has reported ICC values of 0.99([26](#_ENREF_26)).

**Statistical Analysis**

The data analysis will be carried out with the statistical program IBM SPSS for Windows, version 24.0. Armonk, NY: IBM Corp.

The sample size calculation will be carried out using G-power version 3.1.9.7.

Data will be tabulated using percentage distribution and analyzed descriptively by mean, median, mode and graphically presented using bar chart. Depending on normality of data, for within group changes RM-ANOVA or Friedman’s Test and for between group comparison independent t-test or Mann Whitney U test will be used.

A 95% confidence interval will be considered for the difference and statistical significance will be established at p <0.05.

# References

1. Saini V, Guada L, Yavagal DR. Global Epidemiology of Stroke and Access to Acute Ischemic Stroke Interventions. Neurology. 2021;97(20 Suppl 2):S6-s16.

2. Khan MI, Khan JI, Ahmed SI, Ali S. The epidemiology of stroke in a developing country (Pakistan). Pakistan Journal of Neurological Sciences (PJNS). 2019;13(3):30-44.

3. Sherin A, Ul-Haq Z, Fazid S, Shah BH, Khattak MI, Nabi F. Prevalence of stroke in Pakistan: Findings from Khyber Pakhtunkhwa integrated population health survey (KP-IPHS) 2016-17. Pak J Med Sci. 2020;36(7):1435-40.

4. Benjamin EJ, Blaha MJ, Chiuve SE, Cushman M, Das SR, Deo R, et al. Heart Disease and Stroke Statistics-2017 Update: A Report From the American Heart Association. Circulation. 2017;135(10):e146-e603.

5. Smania N, Picelli A, Gandolfi M, Fiaschi A, Tinazzi M. Rehabilitation of sensorimotor integration deficits in balance impairment of patients with stroke hemiparesis: a before/after pilot study. Neurological sciences : official journal of the Italian Neurological Society and of the Italian Society of Clinical Neurophysiology. 2008;29(5):313-9.

6. Balaban B, Tok F. Gait disturbances in patients with stroke. PM & R : the journal of injury, function, and rehabilitation. 2014;6(7):635-42.

7. Batchelor FA, Williams SB, Wijeratne T, Said CM, Petty S. Balance and Gait Impairment in Transient Ischemic Attack and Minor Stroke. Journal of stroke and cerebrovascular diseases : the official journal of National Stroke Association. 2015;24(10):2291-7.

8. Nichols DS. Balance retraining after stroke using force platform biofeedback. Phys Ther. 1997;77(5):553-8.

9. Dong K, Meng S, Guo Z, Zhang R, Xu P, Yuan E, Lian T. The Effects of Transcranial Direct Current Stimulation on Balance and Gait in Stroke Patients: A Systematic Review and Meta-Analysis. Front Neurol. 2021;12:650925.

10. Wist S, Clivaz J, Sattelmayer M. Muscle strengthening for hemiparesis after stroke: A meta-analysis. Annals of physical and rehabilitation medicine. 2016;59(2):114-24.

11. Veerbeek JM, van Wegen E, van Peppen R, van der Wees PJ, Hendriks E, Rietberg M, Kwakkel G. What is the evidence for physical therapy poststroke? A systematic review and meta-analysis. PLoS One. 2014;9(2):e87987.

12. Pollock A, Gray C, Culham E, Durward BR, Langhorne P. Interventions for improving sit-to-stand ability following stroke. The Cochrane database of systematic reviews. 2014;2014(5):Cd007232.

13. van Duijnhoven HJ, Heeren A, Peters MA, Veerbeek JM, Kwakkel G, Geurts AC, Weerdesteyn V. Effects of Exercise Therapy on Balance Capacity in Chronic Stroke: Systematic Review and Meta-Analysis. Stroke. 2016;47(10):2603-10.

14. Bang DH, Cho HS. Effect of body awareness training on balance and walking ability in chronic stroke patients: a randomized controlled trial. J Phys Ther Sci. 2016;28(1):198-201.

15. Tyson SF, Kent RM. Effects of an ankle-foot orthosis on balance and walking after stroke: a systematic review and pooled meta-analysis. Arch Phys Med Rehabil. 2013;94(7):1377-85.

16. Rudd AG, Bowen A, Young GR, James MA. The latest national clinical guideline for stroke. Clinical medicine (London, England). 2017;17(2):154-5.

17. Li Y, Fan J, Yang J, He C, Li S. Effects of transcranial direct current stimulation on walking ability after stroke: a systematic review and meta-analysis. Restorative Neurology and Neuroscience. 2018;36(1):59-71.

18. Comino-Suárez N, Moreno JC, Gómez-Soriano J, Megía-García Á, Serrano-Muñoz D, Taylor J, et al. Transcranial direct current stimulation combined with robotic therapy for upper and lower limb function after stroke: a systematic review and meta-analysis of randomized control trials. Journal of neuroengineering and rehabilitation. 2021;18(1):1-16.

19. Saeys W, Vereeck L, Lafosse C, Truijen S, Wuyts FL, Van De Heyning P. Transcranial direct current stimulation in the recovery of postural control after stroke: a pilot study. Disability and Rehabilitation. 2015;37(20):1857-63.

20. Sohn MK, Jee SJ, Kim YW. Effect of transcranial direct current stimulation on postural stability and lower extremity strength in hemiplegic stroke patients. Annals of rehabilitation medicine. 2013;37(6):759.

21. Elsner B, Kugler J, Pohl M, Mehrholz J. Transcranial direct current stimulation (tDCS) for improving activities of daily living, and physical and cognitive functioning, in people after stroke. The Cochrane database of systematic reviews. 2016;3(3):Cd009645.

22. Navarro-López V, Molina-Rueda F, Jiménez-Jiménez S, Alguacil-Diego IM, Carratalá-Tejada M. Effects of transcranial direct current stimulation combined with physiotherapy on gait pattern, balance, and functionality in stroke patients. A systematic review. Diagnostics. 2021;11(4):656.

23. Carr JH, Shepherd RB. Chapter 3 - Walking. In: Carr JH, Shepherd RB, editors. Stroke Rehabilitation. Oxford: Butterworth-Heinemann; 2003. p. 76-128.

24. Carr JH, Shepherd RB. Chapter 2 - Balance. In: Carr JH, Shepherd RB, editors. Stroke Rehabilitation. Oxford: Butterworth-Heinemann; 2003. p. 35-75.

25. Cuthbert SC, Goodheart GJ, Jr. On the reliability and validity of manual muscle testing: a literature review. Chiropractic & osteopathy. 2007;15:4.

26. Alghadir AH, Al-Eisa ES, Anwer S, Sarkar B. Reliability, validity, and responsiveness of three scales for measuring balance in patients with chronic stroke. BMC neurology. 2018;18(1):141.

**ANNEXURES**

**Anexure 1: Consent form (English)**

**CONSENT FORM FOR PATICIPATION IN A RESEARCH STUDY**

Riphah College of Rehabilitation and Allied Health Sciences, Islamabad.

This research study “Effects of Anodal Transcranial Direct Current Stimulation on Strength in Sub-Acute Stroke” is going to be held in Rafsan Rehabilitation Center Peshawar, Pakistan.

All data collected from will be coded in order to protect your identity and should not be disclosed to anyone. Following the study there will be no way to connect your name with your data. Your answers to the questions will not affect the quality of treatment given to you. If you have any questions or concerns about this study or if any problem arises, please (Dr. Mirza Obaid Baig) at, Riphah College of Rehabilitation and Allied Health Sciences, Islamabad at 092512891832, ext.1198. If you have any question and concern about your rights as a research participant, please contact the Riphah College of Rehabilitation Sciences Review Board at 092512891832, ext.1198.

Your participation in this research study is voluntary. You may not to participate and you may with draw your consent to participate at any time. You will not be penalized in any way should you decide not to participate or to withdraw from this study. In the unlikely event of injury resulting from this research, Riphah College of and Allied Health Science, Islamabad is not able to offer financial compensation nor to absorb the costs of medical treatment. However, assistance will be provided to research subjects in obtaining emergency treatment and professional services that available to the community general at nearby facilities.

My signature below acknowledges my consent to voluntarily participate in this research. Such participation does not release the investigator(s), sponsor(s) or granting agency (ies) from their professional and ethical responsibility to me.

I have read this consent from and have been given the opportunity to ask question. I give my consent to participate in this study

Participant’s signature: ______________________

Date: __________________
